# Composition of the SAGA complex in plants and its role in controlling gene expression in response to abiotic stresses

**DOI:** 10.3389/fpls.2015.00865

**Published:** 2015-10-14

**Authors:** Felipe Moraga, Felipe Aquea

**Affiliations:** ^1^Laboratorio de Bioingeniería, Facultad de Ingeniería y Ciencias, Universidad Adolfo IbáñezSantiago, Chile; ^2^Center for Applied Ecology and SustainabilitySantiago, Chile

**Keywords:** SAGA complex, chromatin remodeling, transcriptional coactivator, abiotic stress, protein complex, histone acetyltransferase

## Abstract

Protein complexes involved in epigenetic regulation of transcription have evolved as molecular strategies to face environmental stress in plants. SAGA (Spt–Ada–Gcn5 Acetyltransferase) is a transcriptional co-activator complex that regulates numerous cellular processes through the coordination of multiple post-translational histone modifications, including acetylation, deubiquitination, and chromatin recognition. The diverse functions of the SAGA complex involve distinct modules that are highly conserved between yeast, flies, and mammals. In this review, the composition of the SAGA complex in plants is described and its role in gene expression regulation under stress conditions summarized. Some of these proteins are likely involved in the regulation of the inducible expression of genes under light, cold, drought, salt, and iron stress, although the functions of several of its components remain unknown.

## Introduction

Transcriptional coactivators are multi-protein complexes that can recognize histone markers, modify chromatin, and recruit the transcriptional machinery to control gene expression (Näär et al., 2001). In general, these complexes regulate eukaryotic gene expression by interacting with transcription factors and/or other regulatory components of the basal transcription machinery. SAGA (Spt–Ada–Gcn5–Acetyl transferase) is a transcriptional coactivator complex involved in the regulation of numerous cellular processes through the coordination of the post-translational modification of various histones. The yeast SAGA complex is thought to control transcription of approximately 10% of genes, particularly stress-related genes (Lee et al., 2000; Huisinga and Pugh, 2004). This complex is generally regarded as a coactivator complex (Kuo et al., 1998), but also has a negative role in gene expression (Belotserkovskaya et al., 2000; Ricci et al., 2002). The SAGA complex is involved in histone acetylation (HAT) (Grant et al., 1997), histone deubiquitination (Daniel et al., 2004), mRNA export (Rodríguez-Navarro et al., 2004), transcription elongation (Govind et al., 2007), chromatin recognition (Pray-Grant et al., 2005), and regulation of the basal transcription machinery (Sterner et al., 1999). Unraveling the modular composition of the SAGA complex has enabled interpretation of its multifunctional role (Wu et al., 2004), principally in regulating the transcription of many stress-inducible (Huisinga and Pugh, 2004) and developmentally regulated genes (reviewed in Wang and Dent, 2014). The diverse functions of SAGA involve the participation of modules that are highly conserved between yeast, flies, and mammals. The SAGA complex is composed of more than 20 polypeptide subunits, grouped in four modules: the deubiquitinating module, the histone acetyltransferase module, and the SPT and TAF modules, which are implicated in the recruitment and SAGA architecture, respectively (Reviewed in Daniel and Grant, 2007 and Koutelou et al., 2010). Despite the abundance of genetic information available for plants, little is known about the presence and role of SAGA in photosynthetic organisms. Recently, a study determined the genes encoding subunits of the SAGA complex across a number of plants species (Srivastava et al., 2015), suggesting conservation of the SAGA complex throughout evolution. The yeast SAGA is particularly important for stress-induced transcription, and this function seems to be conserved during evolution (Spedale et al., 2012). In this review, the composition and our current knowledge of the role of the SAGA complex in the control of gene expression under stress conditions in plants is summarized.

## Histone acetylation module

The histone acetylation (HAT) module contains the General Control Non-depressible 5 (GCN5) acetyltransferase in complex with ADA2, ADA3, and SGF29. This module is completely conserved in several photosynthetic organisms (Table 1). The GCN5 protein, which harbors a HAT domain has been identified in *Arabidopsis thaliana* (Pandey et al., 2002), *Vitis vinifera* (Aquea et al., 2010), and rice (Liu et al., 2012). The GCN5 protein mainly modifies Lys residue 14 in histone H3 in yeast (Kuo et al., 1996; Grant et al., 1999) and Arabidopsis (Benhamed et al., 2006; Earley et al., 2007). HAT by GCN5 has been shown to displace promoter nucleosomes (Barbaric et al., 2001), recruit RNA Polymerase II and coactivators to yeast promoter regions (Qiu et al., 2004; Govind et al., 2005), and increase the efficiency of trimethylation of H3-Lysine 4 in transcribed coding sequences (Govind et al., 2007). In Arabidopsis, GCN5 acetylates not only histones but also other proteins, as ADA2 (Mao et al., 2006), and appears to be a phosphorylated given that a phosphatase physically interacts and dephosphorylates GCN5 *in vitro* (Servet et al., 2008). In addition, GCN5 has a BROMO domain that recognizes acetylated lysine residues and increases the retention of the SAGA complex, promoting its HAT, and other functions (Mujtaba et al., 2007). The presence of the HAT and BROMO domain makes GCN5 a “reader” and “writer” of epigenetic marks. ADA2 (alteration/deficiency in activation 2) is an adaptor protein that physically associates with GCN5 (Grant et al., 1997). In Arabidopsis, two related ADA2 factors (ADA2a and ADA2b) have been identified (Stockinger et al., 2001), but only ADA2b is considered a member of the SAGA complex (Srivastava et al., 2015) Both proteins can bind directly to GCN5 through their N-terminal regions (Mao et al., 2006). This interaction enhances the ability of GCN5 to acetylate histones *in vitro* and enables GCN5 to acetylate nucleosomal histones (Mao et al., 2006). Maize homologs of GCN5 and ADA2 also interact with each other *in vitro* and *in vivo* (Bhat et al., 2003, 2004). SGF29 (SaGa associated Factor 29) is another component of the HAT module. In Arabidopsis, two homologous proteins of yeast SGF29 have been identified (Kaldis et al., 2011). In humans, SGF29 interacts with GCN5 but not with ADA2 (Nguyen-Huynh et al., 2015). Deletion of yeast SGF29 does not affect SAGA integrity or composition of the HAT module, indicating that SGF29 is a peripheral subunit in this complex (Shukla et al., 2012). In addition, SGF29 binds H3K4me2/3 via its double TUDOR domain (Bian et al., 2011), suggesting a critical role in mediating transcriptional regulation through subsequent chromatin modifications. In Humans, ADA3 is associated with GCN5 and ADA2 to form the catalytic module of the SAGA complex and cooperates to stimulate GCN5-mediated HAT of nucleosomal templates (Gamper et al., 2009). There is no evidence of a role for ADA3 in plants.

**Table 1:** **Composition of SAGA complex in plants**.

**Saga Module**	**Yeast**	**Human**	**Physcomitrella**	**Arabidopsis**	**Rice**	**Grapevine**
HAT	GCN5/ADA4	GCN5/PCAF	XP_001766378	GCN5/HAG1	Os10g28040	XP_002275146
	ADA2	ADA2b	XP_001755499	ADA2b (At4g16420)	Os03g53960	XP_002262737
			XP_001784968			XP_002268970
	ADA3	ADA3	XP_001782560	ADA3 (At4g29790)	Os05g28300	XP_002265763
	SGF29	SGF29/STAF36	XP_001755688	SGF29a (At3g27460)	Os12g19350	XP_003633806
			XP_001785583	SGF29b (At5g40550)		XP_003633807
SPT	SPT8	ND	ND	ND	ND	ND
	SPT20/ADA5	SPT20/FAM48A	XP_001762074	SPT20 (At1g72390)	Os01g02860	XP_002272317
	SPT7	STAF65/STAF65γ	XP_001767625	HAF1 (At1g32750)	Os06g43790	XP_010656962
			XP_001779301	HAF2 (At3g19040)		
	SPT3	SPT3	XP_001759999	TAF13 (At1g02680)	Os01g23630	XP_002275358
			XP_001758422			XP_003632409
	ADA1	ADA1/STAF42	XP_001769204	ADA1a (At2g14850)	Os12g39090	XP_002279502
				ADA1b (At5g67410)	Os03g55450	XP_002280562
						XP_002263494
	TRA1	TRRAP	XP_001764071	TRA1a (At2g17930)	Os07g45064	XP_003631895
				TRA1b (At4g36080)		
TAF	TAF5	TAF5L	XP_001769775	TAF5 (At5g25150)	Os06g44030	XP_003631761
						XP_002285276
	TAF6	TAF6L	XP_001762306	TAF6 (At1g04950)	Os01g32750	XP_002276969
				TAF6b (At1g54360)		XP_002264290
	TAF9	TAF9	XP_001785776	TAF9 (At1g54140)	TAF9 (Os03g29470)	XP_002273931
		TAF9b			TAF9b (Os07g42150)	
	TAF10	TAF10	XP_001781637	TAF10 (At4g31720)	Os0926180	XP_002266754
						XP_002267115
	TAF12	TAF12	XP_001781440	TAF12 (At3g10070)	Os01g63940	XP_002277150
				TAF12b (At1g17440)	Os01g62820	
DUBm	UBP8	USP22	XP_001765324	UBP22 (At5g10790)	Os04g55360	XP_002283376
						XP_003633155
	SGF11	ATXN7L3	XP_001779739	SGF11 (At5g58575)	Os05g28370	XP_003632167
			XP_001754483			
			XP_001760795			
	SUS1	ENY2	XP_001759104	SUS1 (At3g27100)	Os01g69110	XP_002269535
			XP_001764723			
	SGF73	ATXN7	XP_001760795	ND	ND	ND
Other subunits	CHD1	ND	XP_001767461	CHR5 (At2g13370)	OsJ_25446	XP_002275100
			XP_001782004			

*ND, Not defined*.

## Recruiting module

This module contains the proteins SPT8, SPT20, SPT7, SPT3, ADA1, and TRA1 and is conserved in several photosynthetic organisms with the exception of SPT8 (Srivastava et al., 2015, Table 1). Notably, orthologs of the *SPT8* gene are also absent in the genomes of metazoans (Spedale et al., 2012). The SPT3 subunit recruits the TATA Binding-Protein (TBP) and contributes to the formation of the preinitiation transcription complex (Dudley et al., 1999). In plants, a homologous protein of SPT3/TAF13 has been described in Arabidopsis (Lago et al., 2004) and pepper (Wen et al., 2013). In Arabidopsis, TAF13 interacts physically with other TAFs (TBP-associated factor) proteins (Lawit et al., 2007) and with MEDEA and SWINGER, both members of a plant variant of Polycomb Repressive Complex 2 (PRC2; Lindner et al., 2013). PRC2 is involved in transcriptional repression through tri-methylation of lys27 of histone H3, suggesting a possible link between SAGA and other complexes involved in chromatin remodeling. SPT20 has a primordial function in the assembly of the SAGA complex, as no intact SAGA could be purified in *spt20* yeast mutant strains (Sterner et al., 1999). In plants, an SPT20 domain containing protein has been reported by Endo et al. (2013) and is an interactor that bridges PHYTCHROME B (phyB) and CONSTANS (CO) proteins involved in the photoperiodic regulation of flowering (Endo et al., 2013). There is no evidence of such a molecular mechanism of SPT20 in plants.

On the other hand, the SPT7 protein works as a scaffold element that maintains and stabilizes the SAGA complex also (Wu et al., 2004). In Arabidopsis, their homologous proteins are HAF1 and HAF2, putative proteins that harbor a histone acetyltransferase, and BROMO domain that can interact with acetylated lysine (Jacobson et al., 2000). The SPT7 BROMO domain interacts weakly with individually acetylated lysine residues (Hassan et al., 2007), suggesting that the BROMO domain within GCN5 is perhaps more important for recognition and binding to acetylated lysine residues in the histone tails, whereas the SPT7 BROMO domain may have another function such as recognition of acetylated transcription factors or multiple lysine residues (Hassan et al., 2007). In Arabidopsis, genetic analysis has shown that HAF2 interacts with GCN5 to integrate light signals, regulating gene expression and growth (Bertrand et al., 2005; Benhamed et al., 2006). Both genes are required for H3K9, H3K27, and H4K12 acetylation on the target promoters (Benhamed et al., 2006).

The SAGA complex is recruited to gene loci by the interaction of yeast TRA1 protein or its mammalian ortholog TRRAP (Transformation/Transcription domain-Associated Protein) with specific transcriptional activators (Brown et al., 2001). These proteins are large and represent almost one quarter of the mass of the entire SAGA complex, suggesting that TRA1 may serve as a scaffold for complex assembly or for recruitment to chromatin in SAGA (Grant et al., 1998; Murr et al., 2007) or other complex (Allard et al., 1999; Knutson and Hahn, 2011). TRA1 and TRRAP proteins show a striking sequence similarity to the family of phosphatidylinositol-3-kinase. There are two genes homologous to TRA1 in Arabidopsis. The genes At2g17930 and At4g36080 encode for a 3858 and 3834 amino acid protein, respectively, with a FAT domain and predicted phosphatidylinositol 3-kinase activity. The recruitment of TRRAP precedes that of GCN5, suggesting that TRA1 and TRRAP function in targeting co-activator complexes to specific promoters during transcription (Memedula and Belmont, 2003). The function of At2g17930, At4g36080 or its homologous proteins in other species of plants has not yet been described.

## Coactivator architecture module

The coactivator architecture of the TAF module contains the TBP-associated factors TAF5, TAF6, TAF9, TAF10, and TAF12. This module is completely conserved in plants (Table 1), and these proteins are shared with the general transcription factor TFIID (Lee et al., 2011). The amino acid sequences of TAFs are conserved from yeast to humans (Struhl and Moqtaderi, 1998; Albright and Tjian, 2000). Initial studies on *in vitro* transcription suggested that TAFs might act as general co-activators that mediate the transcriptional activation of different activators (Goodrich et al., 1996). However, several TAFs have shown tissue- and/or developmental stage-specific expression and are required for the expression of only a subset of genes (Hiller et al., 2001). The endogenous expression of *TAF10* was monitored in transgenic Arabidopsis plants (*pTAF10:GUS*), yielding mostly vascular tissue preferential expression (Tamada et al., 2007). This expression pattern is closely similar to a *TAF10* homologous gene in *Flaveria trinervia*, as been observed by *in situ* hybridization (Furumoto et al., 2005). The *A. thaliana TAF6* gene is expressed in different tissues (Lago et al., 2005). A morphological analysis showed that T-DNA insertion in TAF6 specifically affects pollen tube growth, indicating that this TAF protein regulates the transcription of only a specific subset of genes in plants (Lago et al., 2005). In addition, TAF12 is required for proper hormone response, by negatively regulating cytokinin sensitivity (Kubo et al., 2011) and ethylene response in Arabidopsis (Robles et al., 2007) and TAF5 is an essential gene, required for male gametogenesis, pollen tube growth, and required in transcriptional mechanisms involved in the maintenance of indeterminate inflorescence (Mougiou et al., 2012).

## Deubiquitination module

The Deubiquitination (DUB) module comprises four proteins: UBP8, SGF11, SUS1, and SGF73. This module is conserved in plants with the exception of SGF73 (Table 1). In yeast, the central domain of SGF73 tethers the DUB module to the rest of the SAGA complex while the N-terminal domain forms an integral part of the DUB module (Lee et al., 2009). A homologous protein of SGF73 has only been identified in physcomitrella (Table 1), suggesting that other protein(s) could be involved in this function in higher plants. The homologous protein of UBP8 in Arabidopsis is UBP22 (At5g10790), a member of a family of Ubiquitin-specific proteases highly conserved in eukaryotes. The function of UBP22 has not been described in plants. UBP8 has been described as an ubiquitin protease that specifically removes monoubiquitin from lysine 123 of the H2B C-terminal tail (Henry et al., 2003; Daniel et al., 2004). In humans, biochemical analysis of the substrate specificity of USP22 reveals that it deubiquitylates histone H2A in addition to H2B (Zhang et al., 2008). Although UBP8 contains an ubiquitin-specific hydrolase domain, the protein is inactive unless in complex with the other three DUB module proteins (Weake et al., 2008; Lee et al., 2009). The loci At3g27100 and At5g58575 are the homologous genes of *SUS1* and *SGF11*, respectively. It has been demonstrated that the interaction of SUS1 with the SAGA complex requires UBP88 and SGF11, suggesting that SGF11 could be the direct binding partner of SUS1 (Köhler et al., 2006). Interestingly, although there is no evidence for the role of both proteins in Arabidopsis, the physical interaction between At3g27100 and At5g58575 has been reported (Arabidopsis Interactome Mapping, 2011), suggesting a conserved role inside the SAGA complex in plants.

## Other subunits

The protein CHD1 has been identified as a component of the SAGA complex in yeast (Pray-Grant et al., 2005). This protein is involved in ATP-dependent chromatin remodeling and contains a CHROMO domain that binds methylated H3K4. In Arabidopsis, CHR5 is the homologous protein of CHD1. This gene is expressed during embryo development and seed maturation and is directly involved in the activation of ABI3 and FUS3 expression, key transcriptional regulators of zygotic embryo development (Shen et al., 2015). This protein might recruit SAGA to chromatin and coordinates different complexes implicated in chromatin remodeling, like the COMPASS complex involved in tri-methyl marks on histone 3 lysine4.

## Role of the SAGA complex in control of gene expression under abiotic stress

**Figure 1:**
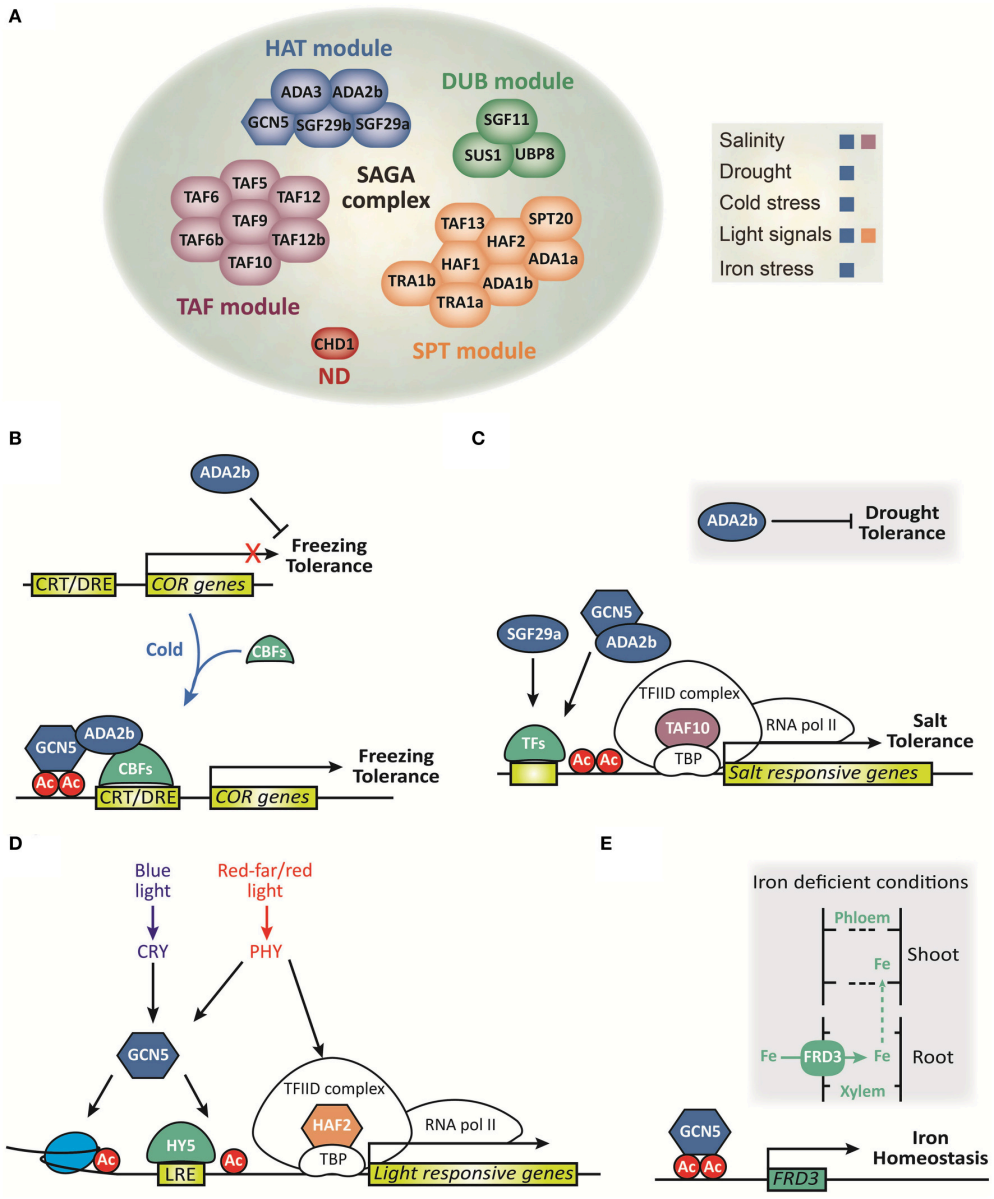
**Composition and function of the SAGA complex in plants. (A)** Schematic representation of each module that integrates into the SAGA complex and its role in abiotic stress response. ND, not defined. **(B–E)** Schematic representation of molecular functions of the SAGA complex under abiotic stress. **(B)** ADA2b represses freezing tolerance before cold exposure. During cold exposure, CBFs are induced and together with ADA2b and GCN5 promotes COR genes induction and consequently freezing tolerance. **(C)** ADA2b represses drought tolerance whereas it promotes histone acetylation of salt stress responsive genes and confers salt tolerance. TAF10 promotes salt tolerance during seed germination, while SGF29a plays a modest role in the expression of salt stress responsive genes in arabidopsis. TFs, transcription factors. **(D)** GCN5 integrates both blue and red/far-red light signals to induce histone acetylation and HY5-dependent gene activation of light responsive genes. TAF1 integrates red/far-red light signals to induce histone acetylation and gene activation of light-responsive genes (adapted from Servet et al., 2010). **(E)** Under deficient iron conditions GCN5 promotes histone acetylation of FRD3, which is involved in the transport of Fe into the xylem, to regulate iron homeostasis.

Plant growth is significantly affected by environmental stresses such as cold, salinity, drought, light quality, temperature, and excess or deficiency of nutrients (reviewed in Mahajan and Tuteja, 2005 and Hänsch and Mendel, 2009). Therefore, plants have developed diverse strategies to adapt their growth in response to environmental changes and ensure reproductive success (Franklin et al., 2005; Bäurle and Dean, 2006). Epigenetic mechanisms have been implicated in regulating the expression of stress related genes (Chinnusamy and Zhu, 2009). Dynamic and reversible HAT under abiotic stress enables the switch between permissive and repressive chromatin that regulates transcription. Different members of the SAGA complex play pivotal roles in the environmental stress response and in many developmental transitions in plants (Figure 1; Chen and Tian, 2007; Vlachonasios et al., 2011; Kim et al., 2015, reviewed in Kim et al., 2010 and Servet et al., 2010). Additionally, the gene expression of some components of the SAGA complex is induced under elevated salt concentration and high temperature, add weight to a potentially significant role of SAGA components gene expression in plants during abiotic stresses (Srivastava et al., 2015).

## Salinity and drought stress

Plants have developed sophisticated signaling pathways that act in concert to counteract salinity and drought stress conditions through the action of transcription factors and histone modifications, thereby promoting the induction of many stress responsive genes and ultimately increasing stress tolerance (Reviewed in Chinnusamy and Zhu, 2009; Huang et al., 2012; Yuan et al., 2013; Golldack et al., 2014). The finding that Arabidopsis have homologs of both *GCN5* and *ADA2* genes (Stockinger et al., 2001) warrant additional study of how HAT-containing complexes related to SAGA complexes activate gene expression under abiotic stress conditions in plants. In Arabidopsis, *ada2b* and *gcn5* mutants, but not *ada2a* mutants, demonstrate pleiotropic effects on plant growth, and development (Vlachonasios et al., 2003). Moreover, both mutants exhibit an altered response to low temperatures and hypersensitivity to salt and abscisic acid (Vlachonasios et al., 2003; Hark et al., 2009). In addition, the whole plant transpiration rate in *ada2b* mutants is lower in comparison to wild-type plants after water starvation, suggesting that drought tolerance arises from a reduction in transpiration water loss that probably occurs through stomata closure (Vlachonasios et al., 2011). Recently SGF29a has been identified as another component of the SAGA complex that is involved in stress response (Kaldis et al., 2011). While in root growth and seed germination assays the *sgf29a-1* mutant plants are more resistant to salt stress, the reduction in transcript levels of salt stress responsive genes compared to wild-type plants suggests that SGF29a plays a modest role in the expression of salt-inducible genes (Kaldis et al., 2011). In contrast, the levels of salt stress responsive genes are dramatically reduced under salinity conditions in *ada2b* mutants. Interestingly, the reduction in transcript levels and the pattern of locus-specific acetylation of histones H3 and H4 of salt stress responsive genes in the *ada2b* mutant plants support the hypothesis that some transcription factors involved in salt stress response are capable of recruiting the SAGA complex to their target promoters (Kaldis et al., 2011).

On the other hand, a mutant screen from a chemical-inducible activation tagging allowed the identification of one mutant, designated stg1 (salt tolerance during germination1), which demonstrates an increased tolerance to salt and osmotic stress in comparison to wild-type plants during seed germination (Gao et al., 2006). *STG1* encodes a putative Arabidopsis TBP-associated factor 10 (TAF10), which constitutes the TFIID complex involved in PIC assembly. The constitutive expression of *TAF10* enhances seed tolerance to salt stress during germination, and the knocked-down mutant is more sensitive to salt stress (Gao et al., 2006). Together, this evidence suggests that TAF10 plays a role in mediating an adaptive response under adverse environmental conditions, but its direct interaction with SAGA complex has not yet been determined.

## Cold stress

The plant adaptive response to cold temperatures involves extensive physiological and biochemical changes such as stabilization of the integrity of cellular membranes and gene expression of inducible cold regulated (COR) genes (Reviewed in Thomashow, 1999; Lissarre et al., 2010). The inducible expression of COR genes is mediated mainly by a family of transcriptional activator proteins known as CBF/DREB1 which recognize the DNA regulatory element CRT/DRE present in the promoters of many COR and dehydration inducible genes (Yamaguchi-Shinozaki and Shinozaki, 1994; Park et al., 2015). The CBF transcription factors alter the expression of more than 100 genes that contribute to enhanced freezing tolerance (Fowler and Thomashow, 2002; Vogel et al., 2005). In Arabidopsis, protein interaction assays revealed that the DNA-binding domain of CBF1 binds directly to ADA2b-containing SAGA complexes (Mao et al., 2006). Additionally, the evidence that the transcriptional activity of Arabidopsis CBF1 in yeasts requires ADA2, ADA3, and GCN5 to activate the transcription of reporter genes carrying the CRT/DRE regulatory element (Stockinger et al., 2001), paired with the observation that the expression of CBFs are induced and COR genes are reduced in *gcn5* and *ada2b* cold-acclimated mutant Arabidopsis plants, supports the notion that CBFs stimulate transcription through recruitment of SAGA transcriptional adaptor complexes to the promoters of COR genes (Vlachonasios et al., 2003). Remarkably, non-acclimated *ada2b* mutant plants are more tolerant to freezing temperatures than wild-type plants, indicating that freezing tolerance in non-acclimated *ada2b* mutant is achieved by a novel, undefined pathway that does not require the expression of CBF or COR genes (Vlachonasios et al., 2003). Thus, ADA2b and GCN5 proteins have similar yet distinct functions in gene expression and may be also components of separate co-activator complexes with different biological activities.

## Light signals

Plants perceive light by a set of wavelength-specific photoreceptors such as phytochromes (PHY) and cryptochromes (CRY) that direct adaptive changes in gene expression in response to environmental signals. Ultimately, these light signals are integrated by downstream DNA-binding transcription factors, which bind to several light responsive elements (LRE) present in the promoters of light-inducible genes (Reviewed in Casal and Yanovsky, 2005; Franklin et al., 2005; Jiao et al., 2007). It has been determined that HAF2 functions in concert with GCN5 to integrate light signals and acetylate the core promoter regions of light-inducible genes (Bertrand et al., 2005; Benhamed et al., 2006). Indeed, double mutations of *HAF2* and *HY5*, a bZIP transcription factor that promotes the expression of light-inducible genes, have a synergic effect on hypocotyl length (a photomorphogenesis trait) and light-activated gene expression under different light wavelengths (Bertrand et al., 2005; Benhamed et al., 2006). This suggests that HAF2 is involved in the signaling pathways of both red/far-red and blue signals, and interacts with HY5 to rapidly activate the expression of light-responsive genes. Moreover, *gcn5/taf1* double mutations result in a further loss of light-responsive genes and exert a cumulative effect on both plant growth and H3K9 acetylation (Benhamed et al., 2006). This evidence, together with the observation that GCN5 and HY5 share many genomic targets (Benhamed et al., 2008), indicates that GCN5 and HY5 might act cooperatively to activate the expression of light-inducible genes. Thus, HAF2 is presumably recruited to its target promoters by interacting with the TBPs, while GCN5 may be recruited to the target promoters by interacting either with specific DNA-binding transcription factors such as HY5 and/or with acetylated histone lysine residues of nearby nucleosomes. Recently, it has been reported that the expression of light-activated genes is considerably reduced in six SAGA subunits in Arabidopsis mutants (Srivastava et al., 2015), indicating that other components of SAGA are involved in the expression of light-inducible genes as well.

## Nutritional stress

Recently a report demonstrated that a mutation in GCN5 resulted in accumulation of manganese, zinc, and iron in the roots (Xing et al., 2015). Specifically, this mutant exhibited impaired iron translocation from the root to the shoot, and this retention was rescued by TSA treatment, a chemical inhibitor of histone deacetylase (Xing et al., 2015). These results suggest that HAT via GCN5 is an important mechanism for iron distribution in Arabidopsis. In addition, GCN5 is necessary for the expression of hundreds of genes involved in iron homeostasis (Xing et al., 2015). These observations, together with the fact that GCN5 directly binds to the promoters of *FRD3*, a key gene in iron homeostasis, and modulates the H3K9/14 global acetylation levels under iron deficient conditions, suggest that GCN5 plays a critical role in iron homeostasis through the regulation of target genes (Xing et al., 2015). There is no evidence for the role of other members of the SAGA complex in the regulation of nutrients homeostasis.

## Concluding remarks

The protein complexes involved in chromatin remodeling and epigenetic modifications are highly conserved in eukaryotes. The SAGA complex is no exception, and although highly conserved in plants, the physical and functional relationships between its different modules remain to be elucidated. Additional study is needed to identify the target genes of the SAGA complex in different environmental conditions and developmental stages, as well as which transcription factors interact with these complexes. Further characterization of the SAGA complex presents the opportunity to identify new actors that participate in the control of gene expression in plants.

### Conflict of interest statement

The authors declare that the research was conducted in the absence of any commercial or financial relationships that could be construed as a potential conflict of interest.
